# SARS-CoV-2 and Multi-Organ damage – What men's health specialists should know about the COVID-19 pathophysiology

**DOI:** 10.1590/S1677-5538.IBJU.2020.0872

**Published:** 2021-03-25

**Authors:** Thiago A. Teixeira, Felipe S. Bernardes, Yasmin C. Oliveira, Mariana K. Hsieh, Sandro C. Esteves, Amaro N. Duarte, Esper G. Kallas, Jorge Hallak

**Affiliations:** 1 Laboratório de Andrologia Clínica e de Pesquisa de Alta Complexidade Centro de Ciência e Inovação em Andrologia São PauloSP Brasil Androscience - Centro de Ciência e Inovação em Andrologia e Laboratório de Andrologia Clínica e de Pesquisa de Alta Complexidade, São Paulo, SP, Brasil; 2 Universidade de São Paulo Divisão de Clinica Urológica Departamento de Cirurgia São PauloSP Brasil Departamento de Cirurgia, Divisão de Clinica Urológica Universidade de São Paulo - USP, São Paulo, SP, Brasil; 3 Universidade de São Paulo Institute for Advanced Studies Men's Health Study Group São PauloSP Brasil Men's Health Study Group, Institute for Advanced Studies, Universidade de São Paulo - USP, SP, Brasil; 4 Universidade Federal do Amapá Faculdade de Medicina Divisão de Urologia AmapáAP Brasil Divisão de Urologia, Faculdade de Medicina, Universidade Federal do Amapá - UNIFAP, Amapá, AP, Brasil; 5 Faculdade de Medicina Ciências da Saúde Albert Einstein Faculdade Israelita São PauloSP Brasil Faculdade Israelita de Ciências da Saúde Albert Einstein, Faculdade de Medicina, São Paulo, São Paulo, SP, Brasil; 6 Clínica de Andrologia e Reprodução Humana CampinasSP Brasil ANDROFERT, Clínica de Andrologia e Reprodução Humana, Campinas, SP, Brasil; 7 Universidade Estadual de Campinas Departamento de Cirurgia CampinasSP Brasil Departamento de Cirurgia, Disciplina de Urologia, Universidade Estadual de Campinas - UNICAMP, Campinas, SP, Brasil; 8 Aarhus University Faculty of Health Aarhus Denmark Faculty of Health, Aarhus University, Aarhus, Denmark; 9 Universidade de São Paulo Departamento de Patologia São PauloSP Brasil BIAS - Grupo de Estudos de Autópsia de Imagens Brasileiras, Departamento de Patologia, Universidade de São Paulo, SP, Brasil; 10 Universidade de São Paulo Departamento de Doenças Infecciosas e Parasitárias São PauloSP Brasil Departamento de Doenças Infecciosas e Parasitárias, Universidade de São Paulo, São Paulo, SP, Brasil; 11 Universidade de São Paulo Departamento de Patologia Unidade de Toxicologia Reprodutiva São PauloSP Brasil Unidade de Toxicologia Reprodutiva, Departamento de Patologia, Universidade de São Paulo, São Paulo, SP, Brasil

## INTRODUCTION

In December 2019, a new RNA coronavirus emerged, named Severe acute respiratory syndrome coronavirus 2 (SARS-CoV-2), and alleged proliferated from the Huanan Seafood Wholesale Market city of Wuhan, in China, to unleash a brutal spreading pandemic with consequences not fully yet understood (1–4). After eleven months, the ensuing coronavirus disease 2019 (COVID-19) stroke 191 countries, infected 100 million-plus, and claimed close to 1 million lives, with an exponential daily increase by tens of thousands worldwide (5). Many health systems from different countries race against time to adjust their care strategies for SARS-CoV-2 infected patients and still maintain non-deferrable procedures in other medical specialties (6–10).

SARS-CoV-2 belongs to the genus Betacoronavirus, which also involves two other zoonotic coronaviruses that provoked epidemics of Severe Acute Respiratory Syndrome (SARS) in 2003 and Middle East Respiratory Syndrome (MERS) in 2012 (11). The Coronavirus family infects humans and other vertebrates and causes deleterious effects on the respiratory, cardiovascular, gastrointestinal, central nervous system, and genitourinary tract (12–14). Studies suggest that severity and mortality of COVID-19 are substantially higher in men than women, drawing fully attention to all Men Health's care professionals (15).

Our proposal is to update Men's healthcare professionals, with the most recent and fast-changing findings on COVID-19 pathophysiology, highlighting some specific multi-organ effects of SARS-CoV-2 infection, primarily in the male genitourinary tract.

### Pathophysiology - General Aspects

Understanding COVID-19 pathophysiology is crucial to comprehend why SARS-CoV-2 is biologically different from SARS-CoV, despite their 80%-plus genome similarities (16). A recent study proposed two plausible scenarios of natural selection for triggering the current pandemic, (i) one beginning in an animal host before zoonotic transfer to humans, and (ii) other starting in humans following zoonotic transfer (17).

For survive and propagate, RNA viruses must balance the capacities for adaptation to new environmental conditions or host cells, whereas maintaining an intact and replication-competent genome. Coronaviruses can make a cross-species jump, with the development of multiple animal coronavirus pathogens (18). Probably, SARS-CoV-2 has derived from bat coronavirus species collected from southwestern China (19).

Since its emergence, SARS-CoV-2 presents higher contagiousness with unprecedented pandemic potential than its predecessors (20). This new disease is clinically asymptomatic for up to five days and remains so for another ten days in 80% of those infected, spreading aggressively but with an elusive and unnoticed behavior (21). Table-1 resumes the COVID-19 clinical manifestations per organ system, categorizing them by disease severity.

**Table 1:** COVID-19 Clinical Manifestations.

Organ systems	Mild disease	Moderate disease	Severe disease
Respiratory	Dry cough Sore throat Rhinorrhea Sneezing	Pneumonia Dyspnea Hypoxemia	Hypoxemia *ARDS
Neurological	Hyposmia-anosmia Hypogeusia-ageusia Visual disturbance Fatigue, somnolence	Headaches Nausea Emesis Dizziness Myalgia Ataxia Encephalopathy	Large-vessel strokes Seizures Meningoencephalitis Neuropathy Guillain-Barré Syndrome Neurogenic *ARDS Coma
Gastrointestinal	Nausea, Emesis Diarrhea Heartburn	Loss of appetite Abdominal pain Distension	Hematemesis Melena
Cardiovascular	Chest pain Arrhythmia Sinus tachycardia Blood coagulation	Myocarditis Arterial or venous thromboembolism §CRS	Cardiomyopathy Acute heart failure Pulmonary embolism +DIC
Urinary	Proteinuria Hematuria	#AKI	#AKI
Testis	Seminal abnormalities	Scrotal discomfort Epididymitis Orchitis Seminal abnormalities	Orchitis Subfertility Infertility? Transient /Prolonged? Hypogonadism

Legends:

* **ARDS** = Acute respiratory distress syndrome

§ **CRS** = Cytokine release syndrome

+ **DIC**: Disseminated intravascular coagulation

# **AKI** = Acute kidney injury **References**: (1, 2, 12–15, 20, 21, 33, 34, 36, 66)

### Mechanism of host invasion

SARS-CoV-2 is an enveloped, positive-sense, single-stranded RNA virus (11), whose life cycle within the host consists of five acts: attachment, penetration, biosynthesis, maturation, and release (22). During the attachment phase, SARS-CoV-2 interface with the angiotensin-converting enzyme 2 (ACE2), a membrane receptor expressed on the surface of not exclusively airway epithelial cells, but also in the testis, kidneys, and in the heart (23, 24). Notably, the ACE2 receptor plays a critical role in the pathogenesis of COVID-19 as it determines viral entry in human cells (25).

ACE2 is an enzyme that physiologically acts as a receptor for cell entry of both the SARS virus, also activating the Renin-Angiotensin-Aldosterone System (RAAS), a complex network of critical interconnecting cascades of vasoactive peptides common to a multitude of biological systems and ultimately responsible for the tonus of the vascular system and essential for adequate endothelial functions (26).

The high SARS-CoV-2 infectiousness in the context of ACE2 receptors relies on understanding the virus's structure and ligand properties. In the case of SARS-CoV, the spike glycoprotein (S protein) on the virion surface mediates receptor recognition and membrane fusion (27). During viral infection, the subunit S1 of the S protein, which contains the receptor-binding domain (RBD), directly binds to the peptidase (PD) domain of ACE2, whereas the S2 subunit is responsible for membrane fusion. Therefore, the primary physiological purpose of ACE2 in the maturation of angiotensin is replaced in full obedience to the virus program (16).

A notable feature of the SARS-CoV-2 genome is that it appears to be engineered for optimization of binding to the human receptor ACE2, and its S protein has a functional polybasic (furin) cleavage site at the S1-S2 boundary through the insertion of 12 nucleotides. Additionally, the acquisition of three O-linked glycans around the site, inexistent in previous SARS-CoV, confers high bounding capacity to this new version (17). After ACE2 engagement, SARS-CoV-2 employs the transmembrane protease serine 2 (TMPRSS2) for S protein priming, contributing to virus binding and indispensable for cell invasion (25). SARS-CoV-2 mechanism of human cell invasion is unique and has not been described in any other known coronaviruses (1).

### Cytokine Release Syndrome and SARS-CoV-2 immunopathology

Cytokine-release syndrome (CRS), also known as a cytokine storm, represents an excessive proliferation of immune cells resulting in an enhanced inflammatory cytokine release, tissue damage, and ultimately multi-organ and system failure (28). In SARS-CoV-2 infected patients, cytokines such as interleukin (IL)-1, IL-6, IL-10, tumor necrosis factor-alpha (TNF- α), and interferon-gamma (IFN- γ), are elevated (29, 30). Notably, higher IL-6 and IL-10 levels have prognostic value for the disease, since they disclose a positive association with worse severity of the infection (29). Also, some cytokines, as IL-1β, IL-6, and TNF, may contribute to the augmented risk of coagulopathy during the infection, since they inhibit the protein-C-system, the tissue factor, and the antithrombin-mediated inhibition of thrombin (31).

Besides CRS, two other mechanisms seem to contribute to the multi-organ dysfunction found in COVID-19, the T-cell dysregulation, and the hemophagocytic lympho-histiocytosis (sHLH) (29, 32). Despite the hyperactivated state of CD4+ and CD8+ T cells, decreased levels of these same cells in peripheral blood have been reported (33, 34). The virus infects and destroys T cells, eliciting a deep lymphopenia, further aggravated by the inflammatory viral response that damages lymphopoiesis and increases lymphocyte apoptosis (35). On the other hand, the sHLH consists of a misregulated positive feedback loop between immune cells and cytokines, provoking tissue damage, and multi-organ failure (32).

In the end, the proposed mechanisms of CRS, sHLH, and T-cell dysregulation morbidity elicit organ-system failure syndromes, such as acute respiratory distress syndrome (ARDS) or acute kidney injury (AKI), which by themselves increase the mortality rates in severe cases.

### SARS-CoV-2 and multi-organ damage

SARS-CoV-2 pathogenesis has several determinants to severe damage, firstly in the lungs and then with systemic dissemination. Table-2 summarizes the current pathological findings in COVID-19.

**Table 2:** COVID-19 Pathological Features.

Organ systems	Microscopic findings
Respiratory	*DAD Lymphocytic infiltration in interstitial regions Lung epithelial cell death by #NETs Intra-alveolar fibrin deposition Exudate formation Viral particles within pneumocytes Hyaline membrane formation Cytoplasmic vacuolization in pulmonary arteries Megakaryocyte with multinuclear appearance within the branching small vessels Pulmonary microangiopathy Small vessel thrombosis with alveolar hemorrhage Thickening of alveolar capillaries Bronchial epithelial denudation Loss of cilia Squamous metaplasia
Neurological	Edema Hyperemia Reactive gliosis Detectable viral particles Hemorrhagic white matter Axonal injuries Leukocytic infiltration §ADEM-like appearance Neocortical microscopic infarcts No infiltration of inflammatory cells or neural cell degeneration
Gastrointestinal	Lymphocytes infiltration in the esophageal squamous epithelium and lamina propria of the stomach, duodenum and rectum Interstitial edema Viral nucleocapsid protein in glandular epithelial cell of stomach, duodenum and rectum Viral particles in infected patients’ stools
Cardiovascular	Myositis Mild lymphomononuclear myocarditis Fibrin microthrombi Cardiomyocyte hypertrophy Myocardial fibrosis Myocyte necrosis near to lymphocytes Infiltration of mononuclear leukocytes in interstitial areas Endotheliitis
Urinary	Proximal +ATI Luminal brush border sloughing Vacuole degeneration Tubular necrosis Infiltration of lymphocytes and macrophages Interstitial fibrosis in cortical parenchyma ∞MAC deposition in tubules Mild focal tubular atrophy Hypertrophy and hyperplasia of glomerular epithelial cells Hemosiderin pigment granules in tubular epithelium Podocyte vacuolation Cellular swelling in infected renal tissues &SER and &RER dilation Infiltration of inflammatory cells in the arcuate artery Dilated and swollen capillary vessels in glomeruli Segmental fibrin thrombus in glomerular capillary loops Endothelial hyperplasia Foamy-like appearance of endothelial cells
Testis	Orchitis with fibrin microthrombi Mild lymphocytic inflammation Reduced Leydig cell population Substantial seminiferous tubular damage

Legends:

* **DAD** = Diffuse alveolar damage

# **NETs** = neutrophil extracellular traps

§ **ADEM** = Perivascular acute disseminated encephalomyelitis

+ **ATI**: Proximal acute tubule injury

∞ **MAC**: Membrane attack complex **&SER**: Smooth endoplasmic reticulum; **&RER** = Rough endoplasmic reticulum **References:** (14, 15, 23, 35, 38–42, 47, 48, 67).

### Lungs

In humans, SARS-CoV-2 usually accesses the airways and invades the alveolar space tissue, whose alveolar epithelial type II cells positively express ACE2 and TMPRSS2 (36). Later in the infection, the virus infects pulmonary capillary endothelial cells, stimulating neutrophils and monocyte's migration. Interstitial mononuclear inflammatory cells infiltrate, causing alveolar space's edema with early-onset intense hyaline membrane formation (35).

Autopsies confirmed the scenario of proliferative and exudative diffuse alveolar damage (DAD). Diffuse alveolar exudates express exudative DAD with septal edema, hyaline membranes, and mild to moderate lymphocytic infiltration. Simultaneously, proliferative DAD is described by a scarce, organized fibrous tissue within alveolar lumen and septa and is more prevalent in patients with a prolonged hospitalization period (37).

Neutrophil extracellular traps (NETs) are essential mediators of tissue damage in immune-mediated events such as COVID-19. NET's release by viral-activated neutrophils promotes lung epithelial cell death in severe SARS-CoV-2 infection (38).

Coagulation cascade activation and clotting factors consumption occur in severe cases, with the consequent microthrombi formation in pulmonary and systemic arteries, resulting in a pulmonary ventilation-perfusion mismatch and peripheral ischemic events in critically ill patients (37, 39).

### Heart

As ACE2 is highly expressed in the cardiovascular system, the probability of developing heart injury during SARS-CoV-2 infection is proportionally high. The virus interaction with ACE2 triggers a signal that unleashes a disrupted immunologic response, the Cytokine storm mentioned above, which probably be responsible for the cardiac damage (40).

Some cardiovascular findings in autopsy studies remain with undetermined etiology, such as myositis, mild lymphomononuclear myocarditis, and fibrin microthrombi. These lesions can be caused by the direct action of the virus, systemic inflammation, or shock. Other findings, such as cardiomyocyte hypertrophy and myocardial fibrosis, are related to patient's comorbidities, like diabetes and hypertension (37).

### Intestines

Absorptive enterocytes from the ileum and colon positively express ACE2 receptors, specifically at the villous brush border, in smooth muscle cells of the intestinal muscular layers and vascular smooth muscle cells and endothelium (41). Besides proteomic research shows that ACE2 was enhanced in inflammatory bowel diseases, there is no evidence for increased susceptibility for SARS-CoV-2 infection in patients with these comorbidities (42).

A systematic review and meta-analysis of 10.890 patients reported a prevalence of up 10% of gastrointestinal symptoms, such as nausea, vomiting, abdominal pain and diarrhea, and up to 20% of abnormal liver enzymes in COVID-19 patients (43). SARS-CoV-2 particles are found in infected patient's stools, and further research is mandatory to elucidate the usefulness of this finding in clinical management and disease transmission chain (44).

### Brain

In the animal model, SARS-CoV invades the brain primarily via the olfactory bulb, and consequently, transneural viral spread, and probably SARS-CoV-2 follows this neuroinvasion pathway (45). Additionally, ACE2 expression in cerebral vascular endothelium and subsequent endothelial damage could drive the virus another alternative brain pathway (46).

Olfactory bulb invasion is responsible for anosmia and dysgeusia, while injured neurons within the respiratory center in the brainstem may be partially responsible for respiratory symptoms in some patients (47). Autopsy specimens from different studies demonstrated that brain tissue of COVID-19 patients presents with intense edema, hyperemia, cerebral small-vessel disease, reactive gliosis, and detectable viral particles (32, 37, 48).

### Kidneys

In the urinary system, the kidneys represent the primary target organ for COVID-19 because of the up-regulated ACE2 and TMPRSS2 expression, chiefly in the proximal tubular cells and, on a smaller scale, in podocytes (36, 49, 50). The prevalence of acute kidney injury (AKI) among infected patients is low and varies according to the severity of the disease, ranging in different studies from 0.5% to 7.0% in the overall analysis and from 6.0% to 25% in patients who needed intensive care support or died (32, 51–53).

The pathophysiologic mechanisms underlying AKI in COVID-19 patients remain unclear, but three possibly interconnected processes seem to be implicated, expressly the mentioned CRS, some systemic deleterious metabolic alterations, and a multi-organ cross-talk damage (54). CRS can induce a cardio-renal syndrome type 1, marked by intra-renal inflammation, cardiomyopathy, increased vascular permeability, and volume depletion, mediated predominantly by the pro-inflammatory IL-6 (28). Factors that probably intensify cytokine generation in CRS are invasive mechanical ventilation, continuous kidney replacement therapy (CKRT), and extracorporeal membrane oxygenation (ECMO) (54).

Critically ill patients frequently develop systemic complications, such as secondary infections, hyperkalemia, metabolic acidosis, and rhabdomyolysis, contributing to hemodynamic instability and, consequently, to AKI genesis (54). During the SARS-CoV-2 infection, two organ cross-talk axes possibly involved in AKI pathophysiology are the Lung-kidney and the Heart-kidney loops. In the Lung-kidney cross talk, infected patients with the severe acute respiratory syndrome (SARS) produce higher levels of IL-6, which are associated with pulmonary hemorrhage, medullary hypoxia, and tubular cell injury (55). During the CRS cardiomyopathy, characteristic of the Heart-kidney cross-talk, renal vein congestion, hypotension, and, consequent, decrease in glomerular filtration rate are also contributing factors to AKI development (54).

In a systematic review with 11 studies (n=195 patients), just 5.74% (95% confident interval 2.88-9.44%) tested positive for SARS-CoV-2 RT-PCR in urine (56). Direct invasion of the urinary system and CRS-induced renal dysfunctions could be responsible for SARS-CoV-2 shedding in urine (57).

### Testis and Semen

In the male reproductive tract, the testis had the highest ACE2 density (58, 59), and the receptor is widely expressed in Leydig, Sertoli-cells and spermatogonia (60). In humans, ACE2 acts as a physiological modulator for steroidogenesis and a regulator of reactive oxygen species (ROS) formation, probably affecting the germ cell lineage (61, 62). ACE2 receptors have a strong influence on male reproductive function since fertile men have higher ACE2 levels than infertile subjects with severe spermatogenesis impairment (63).

Few reports demonstrated the testicular involvement during SARS-CoV-2 infection. In a case series of infected men with mild-to-moderate symptoms, almost 18% denounced a scrotal discomfort around the time of diagnosis (64). Simultaneously, a report described a case of orchiepididymitis in the setting of confirmed SARS-CoV-2 infection in a 14-year-old boy, whose clinical evolution happened without classical respiratory symptoms (65).

Autopsy studies displayed testicular findings such as orchitis with fibrin microthrombi, mild lymphocytic inflammation, reduced Leydig cell population, and a substantial seminiferous tubular damage (37, 66). In situ hybridization has failed, at the moment, to find both SARS-CoV and SARS-CoV-2 in the testicular tissue (50, 66–68), while there is a report of SARS-CoV-2 particle detection in one testis specimen by real-time polymerase chain reaction (RT-PCR), possibly associated with high viral load (66). A testicular specimen analyzed by transmission electron microscopy (TEM) recently demonstrated the presence of SARS-CoV-2 in a single autopsy of an infected COVID-19 patient (69).

Regarding the impact of COVID-19 on endocrine intratesticular function, an enhancement in luteinizing hormone (LH) and consequent decrease in total testosterone/luteinizing hormone (T/LH) ratio in infected men was reported (69). This decreased T/LH ratio described was correlated to higher levels of C-reactive protein and higher values of white blood cell count, possibly meaning a transient stage of hypogonadism, to be further confirmed by future research (70).

There is no current evidence of SARS-CoV-2 sexual transmission, and just in one study, the virus was detected by RT-PCR in six of 38 semen samples, two of which collected during the convalescent period (71). However, most studies fail to demonstrate the virus in the semen in acute and convalescent stages of COVID-19 (64, 69, 70, 72). Due to difficulties in collecting semen samples in the acute phase of the disease and due to the short expression of the virus in the body, the presence or absence of the SARS-CoV-2 in the ejaculate must be considered still an open question.

## CONCLUSIONS

The huge coronavirus family has been around for millennia and probably has infected Humanity many times in the past. XXI century facilities in transportation and increased population densities in the urban environment worldwide have given viruses comfortable easy-ride and increased chances for survival and reproduction, the ultimate desire of any living organisms. The downside of this evolutionary step forward in infectiousness capability is that Humans have not adapted to increased awareness for this new battlefield. We were caught by surprise as in the first SARS-CoV infection in 2003, scientists were not given the right opportunity and incentives for research, and now Humanity is paying a high price. The pathophysiological consequences are largely still unknown. Men are more susceptible to developing more severe outcomes, including death, than women, which drives the attention to our field of Men's Health specialists. Our goal is to alert and increase scientific knowledge, awareness, and preparedness for future outbreaks.
